# Pituitary Stalk Interruption Syndrome: A Case Series

**DOI:** 10.7759/cureus.84437

**Published:** 2025-05-19

**Authors:** Binod Prusty, Devadarshini Sahoo, Sambit Das, Dayanidhi Meher, Vishal Agarwal, Sandeep K Sahu, Arun Choudhury, Bijay Das

**Affiliations:** 1 Endocrinology, Diabetes and Metabolism, Kalinga Institute of Medical Sciences, Bhubaneswar, IND; 2 Endocrinology, Diabetes and Metabolism, Kalinga Institute of Medical Sciences, Bhubaneshwar, IND

**Keywords:** growth hormone deficiency, hypogonadotropic hypogonadism, hypothyroidism, pituitary stalk interruption syndrome, short stature

## Abstract

Pituitary stalk interruption syndrome (PSIS) is a rare congenital endocrine condition characterized by a developmental anomaly of the pituitary gland, leading to deficiencies in anterior pituitary hormones due to the absence or underdevelopment of the pituitary stalk and anterior pituitary gland. An ectopic posterior pituitary; anterior pituitary hypoplasia or aplasia; and a thin, interrupted, or missing pituitary stalk constitute the classic triad of symptoms that define PSIS. PSIS is manifested in various forms and may be identified at different stages of life. PSIS may present as an isolated growth hormone deficiency occurring due to the deficiency of multiple pituitary hormones. Although the exact aetiology remains unknown, genetic mutations are considered a potential causative factor for disease onset. Hormone replacement therapy and early detection of PSIS are essential for preventing long-term consequences. Here, we report three cases in which patients at different stages of life presented with a wide variety of clinical manifestations of PSIS.

## Introduction

Pituitary stalk interruption syndrome (PSIS) was first reported in 1987 by Fujisawa et al. in a patient with idiopathic pituitary dwarfism following surgical resection of the pituitary stalk [1]. The incidence of PSIS is estimated to be 0.5 per 100,000 live births [2]. PSIS has a variable age of presentation, ranging from early childhood to adulthood, and is associated with characteristic features of anterior pituitary hormone deficiencies. PSIS presenting as an isolated growth hormone deficiency in early childhood may gradually progress to multiple pituitary hormone deficiency (MPHD) during adulthood. MPHD is defined as the deficiency of more than one pituitary hormone that can be due to acquired, idiopathic, or heritable factors [3].

PSIS must be distinguished from other structural and functional causes of hypopituitarism, particularly those presenting with overlapping clinical and radiological features like septo-optic dysplasia, where central hypothyroidism (70%) followed by growth hormone deficiency (55%) is seen [4]. Other differential diagnosis includes isolated growth hormone deficiency, congenital hypopituitarism due to *PROP1*, *HESX1*, *LHX3*/*LHX4 *mutations, and craniopharyngioma. Hypoglycaemia and failure to thrive are the main complaints when PSIS presents at birth, whereas growth retardation and delayed puberty are the primary concerns when it presents during childhood and adolescence to early adulthood, respectively [5].

The exact aetiology of PSIS remains unknown. Various hypotheses have been proposed, some of which suggest an association with adverse perinatal events such as breech delivery and hypoxia. Recent data suggest that genes involved in defective pituitary development are also responsible for PSIS. Diagnosis of PSIS is primarily based on neuroimaging and endocrine evaluation. Magnetic resonance imaging is the key for the diagnosis of PSIS, characterized by the clinical triad of a thin pituitary stalk, an ectopic posterior pituitary gland, and hypoplasia or aplasia of the anterior pituitary gland [6]. Hormonal assessment including testing for growth hormones, thyroid stimulating hormone, adrenocorticotropic hormone, and gonadotropin hormone, and since hormone deficiencies may evolve; hence, serial evaluation is also necessary. 

Genetic testing is recommended in syndromic cases or familial hypopituitarism to look for mutations in *SOX3*, *OTX2*, *HESX1*, and *LHX4*,* *which have been associated with PSIS. Management of PSIS includes timely replacement of deficient hormones along with regular follow-up so as to detect the emergence of newer hormone deficiencies. Inappropriate diagnosis and delayed treatment not only affect growth and gonadal development but also can cause metabolic complications like dyslipidemia, nonalcoholic fatty liver, nonalcoholic steatohepatitis, and hyperuricemia [7,8,9]. PSIS, as a rare cause of pituitary dwarfism and multiple pituitary hormone deficiency, should be taken into consideration, as close monitoring is essential for long-term prognosis. Here, we report three patients presenting PSIS at different ages and with different clinical manifestations.

## Case presentation

Case 1

An 11-year-old girl presented at the endocrinology outpatient department with the primary complaint of inadequate weight and height gain over the past 6 years. She was born to non-consanguineous parents. The girl was a full-term baby delivered vaginally in a breech position, with no unfavorable prenatal findings. She cried immediately after birth and had a birth weight of 3.4 kg. There was no history of prolonged jaundice, failure to thrive, or any episodes of hypoglycaemia. Her academic performance was average, and she had no history of delayed developmental milestone achievement. She had no history of chronic systemic illness, head trauma, meningitis/encephalitis, headache, or visual defects. On examination, she had severely short stature, with height 108.9 cm, a height standard deviation score of -4.86, a height age of 5 years 3 months, an upper to lower segment ratio (calculated by dividing the length of the upper segment (from the pubic symphysis to the top of the head) by the length of the lower segment (from the pubic symphysis to the floor)) of 0.89, weight 16.3 kg, a weight standard deviation score of -3.89, and a weight age of 5 years. She had a cherubic face with a depressed nasal bridge and midfacial hypoplasia. Sexual maturity rating (SMR) staging showed Tanner stage 1 with normal female external genitalia. On systemic examinations, heart and breath sounds were normal.

The patient’s calcium profile, electrolyte levels, renal and liver function tests, complete blood count, and IgA tissue transglutaminase levels (coeliac serology) were normal (Table 1).

**Table 1:** The basic metabolic panel and complete blood count results for the three patients in this case series. AST, aspartate aminotransferase; ALT, alanine aminotransferase; tTG , tissue transglutaminase; IgA, immunoglobulin type

Parameters	Case 1	Case 2	Case 3	Reference
Haemoglobin (gm/dl)	13.5	12.9	13.1	11.5-15.5
Total leucocyte count/ µl	5600	7800	6760	4000-10000
Total Platelet count x 10^3^/µl	310	243	339	150-410
Serum Creatinine (mg/dl)	0.81	0.79	0.9	0.7-1.3
Serum Urea (mg/dl)	16	21	19	12-42
Serum Sodium (mmol/l)	138	136	139	135-145
Serum Potassium (mmol/l)	3.8	4.1	3.7	3.5-4.1
Serum Calcium (mmol/l)	8.9	8.8	9.1	8.6-10.3
Serum Phosphorus (mmol/l)	1.1	1	1.1	0.8-1.45
AST (U/L)	15	21	19	0-40
ALT (U/L)	10	14	20	5-40
Anti-tTG IgA (U/mL)	1.38	1.97	1.64	Positive: >= 20 Negative: < 20
Urine analysis	Normal	Normal	Normal	
Arterial blood gas analysis	Normal	Normal	Normal	

The hormonal profile (Table 2) revealed growth hormone deficiency accompanied by hypothyroidism.

**Table 2:** Hormonal evaluation findings and their reference ranges for the three patients in this case series TSH, thyroid-stimulating hormone; LH, luteinizing hormone; FSH, follicle-stimulating hormone; ACTH, adrenocorticotrophic hormone; IGF1, insulin-like growth factor 1; FT4, free thyroxine

Parameters	Case 1	Case 2	Case 3	Reference
TSH (mIU/L)	2.915	0.3	7.19	0.27-4.2
FT4 (pmol/L)	7.46	7.98	3.86	10.29-25.74
IGF1 (nmol/L) (Age matched normal range)	2.49 (12.55-72.42)	3.59 (7.45-55.69)	2.61 (7.45-55.69)	
8 am Cortisol (nmol/L)	621.51	94.89	87.72	184.82-623.44
ACTH (pmol/L)	7.3	0.33	0.73	1.58-14
LH (IU/L) (Age-matched normal range)	1.56 (0.02-4.1)	0.2 (0.7-7.2)	<0.07 (0.95-5.6)	
FSH (IU/L) (Normal range)	6.06 (0.7-7.3)	0.28 (0.8-8.2)	0.50 (1.5-14.3)	
Testosterone (nmol/L)		0.034	0.34	8.5-31.8
Prolactin (µ/L)	9.2	11.8	10.8	3.6-12

**Figure 1:**
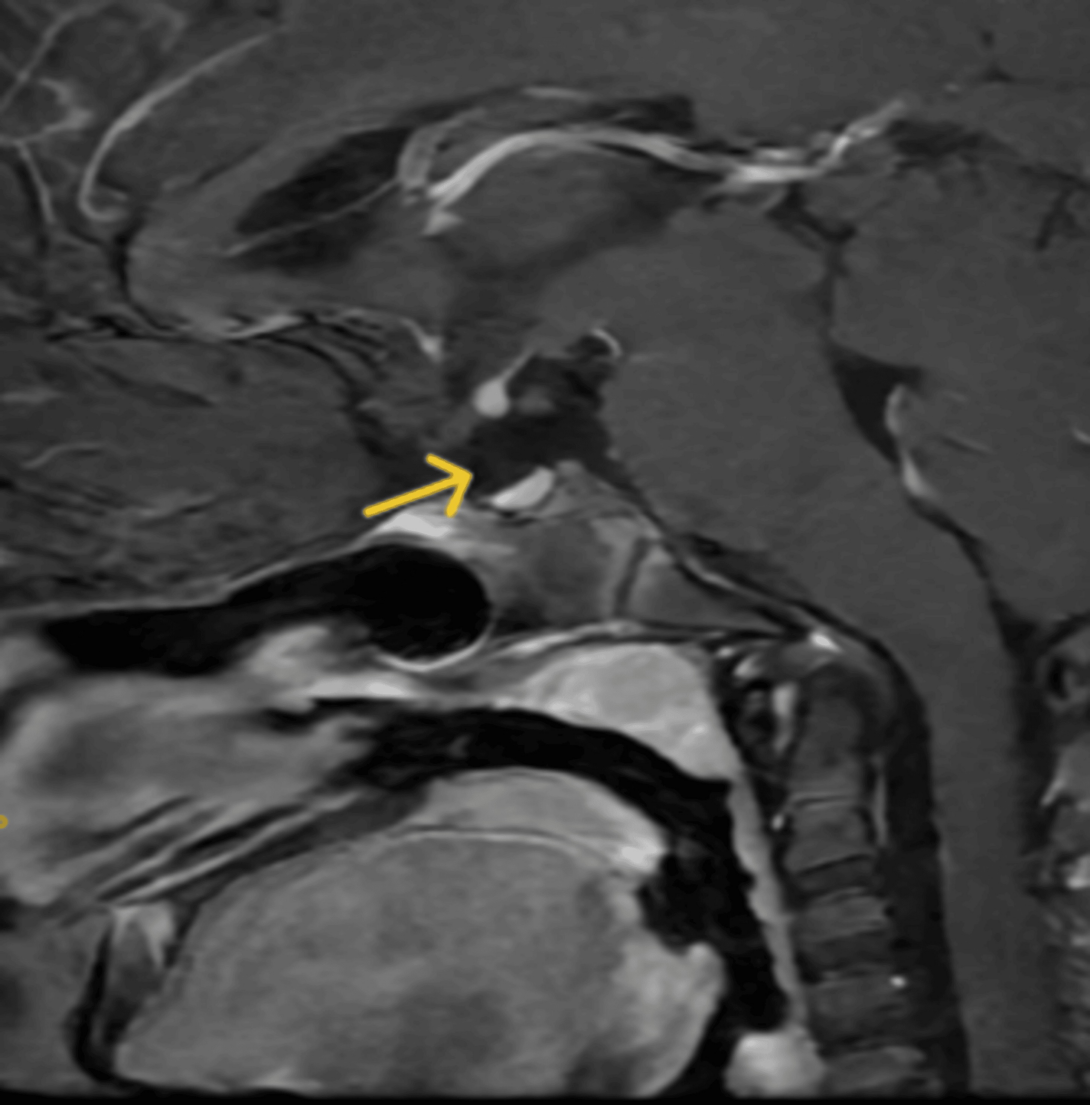
Sagittal T1-weighted contrast-enhanced magnetic resonance imaging of the sella turcica shows absent pituitary infundibulum (yellow arrow) in Case 1

**Figure 2:**
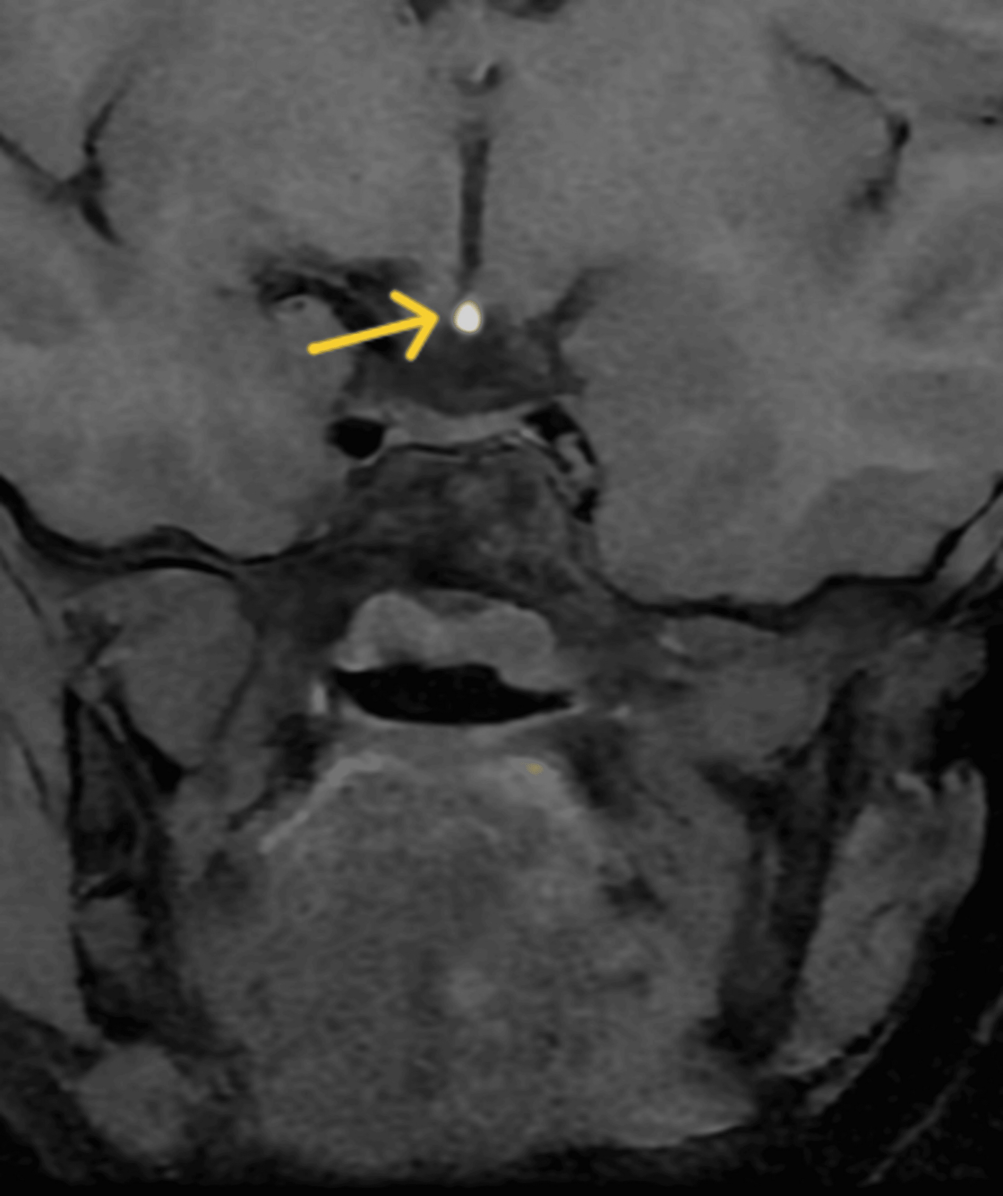
Contrast-enhanced magnetic resonance imaging of the sella turcica of Case 1 Contrast-enhanced magnetic resonance imaging of the sella turcica showing an ectopic posterior pituitary (yellow arrow) with a hypoplastic anterior pituitary measuring 2.3 mm in height, 5.4 mm in the anteroposterior dimension, and 8.1 mm in coronal diameter, and absent/thin pituitary infundibulum.

The patient's bone age was 6 years (<-2.5 SD for age). Contrast-enhanced magnetic resonance imaging (CEMRI) of the sella turcica showed an ectopic posterior pituitary with a hypoplastic anterior pituitary measuring 2.3 mm in height, 5.4 mm anteroposteriorly, and 8.1 mm in coronal diameter, and absent pituitary infundibulum (Figure 1 and Figure 2).

On the basis of these findings, the patient was diagnosed with PSIS with MPHD (growth hormone deficiency (GHD) and thyroid-stimulating hormone (TSH) deficiency) [3]. Since TSH and free thyroxine (T4) have a log-linear relationship, even a slight decrease in free T4 levels usually results in a large increase in TSH, but in this case in the presence of low free T4, the serum TSH is still in normal range, which signifies inadequate response, and hence, the patient was diagnosed with central hypothyroidism or TSH deficiency [10,11,12]. In PSIS, GHD can occur despite normal adrenocorticotropic hormone (ACTH), follicle-stimulating hormone (FSH), luteinizing hormone (LH), and prolactin levels, especially early in the disease course, which can deteriorate over time [13], as seen in this case. The patient was managed with levothyroxine 2.3 mcg/kg/day [14], followed by recombinant human growth hormone administered at a dose of 0.19 mg/kg/week. After 3 months, her thyroid function normalized, and she showed a height gain of 3 cm. During this period, she also developed adrenal insufficiency and was treated with hydrocortisone (5 mg/day). The subsequent follow-up showed adequate height with a growth rate of 10 cm/year. PSIS is a gradually evolving disease that can present as isolated growth hormone deficiency or multiple pituitary hormone deficiencies [15]; therefore, serial monitoring is necessary to identify the hormone deficiency in the future, as seen in this case.

Case 2

A boy aged 15 years and 7 months presented with the chief complaint of poor height and weight gain in the last 11 years. He was born from a non-consanguineous marriage. He was born at term by vaginal breech delivery, with no adverse pregnancy outcomes. His birth weight was 2.8 kg, and he cried immediately after birth. He had no history of prolonged jaundice, failure to thrive, or episodes of hypoglycemia. He achieved normal developmental milestones on time and showed good academic performance. There was no history of chronic systemic illness. Physical examination revealed severely short stature: height 119 cm (<3rd percentile), a height standard deviation score of -6.19, a height age of 6 years 7 months, an upper-to-lower segment ratio of 0.98, weight 20kg (<3rd percentile), a weight standard deviation score of -2.93, with a weight age of 6 years and 3 months, and no midline defects, and bilateral testicular volume was 2 ml. The stretched penile length was 6 cm, which was less than -2.5 SD for his age, and SMR staging indicated Tanner stage 1. The systemic examination was normal.

**Figure 3:**
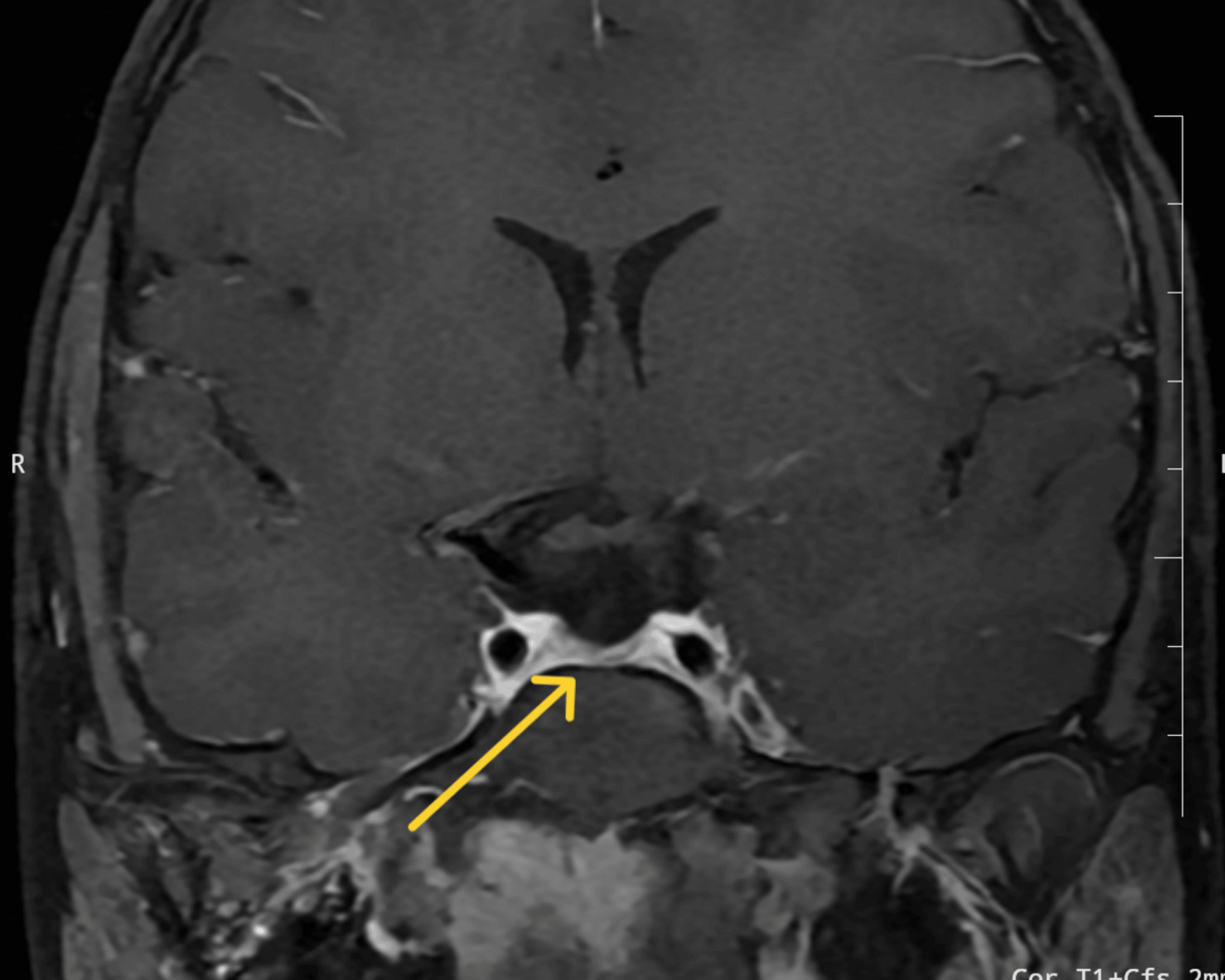
Contrast-enhanced magnetic resonance imaging of the sella turcica of Case 2 Contrast-enhanced magnetic resonance imaging of the sella turcica showing an ectopic posterior pituitary with a hypoplastic anterior pituitary (yellow arrow) measuring 1.9 mm in height, 4.6 mm in the anteroposterior dimension, and 6.8 mm in coronal diameter, and absent pituitary stalk.

**Figure 4:**
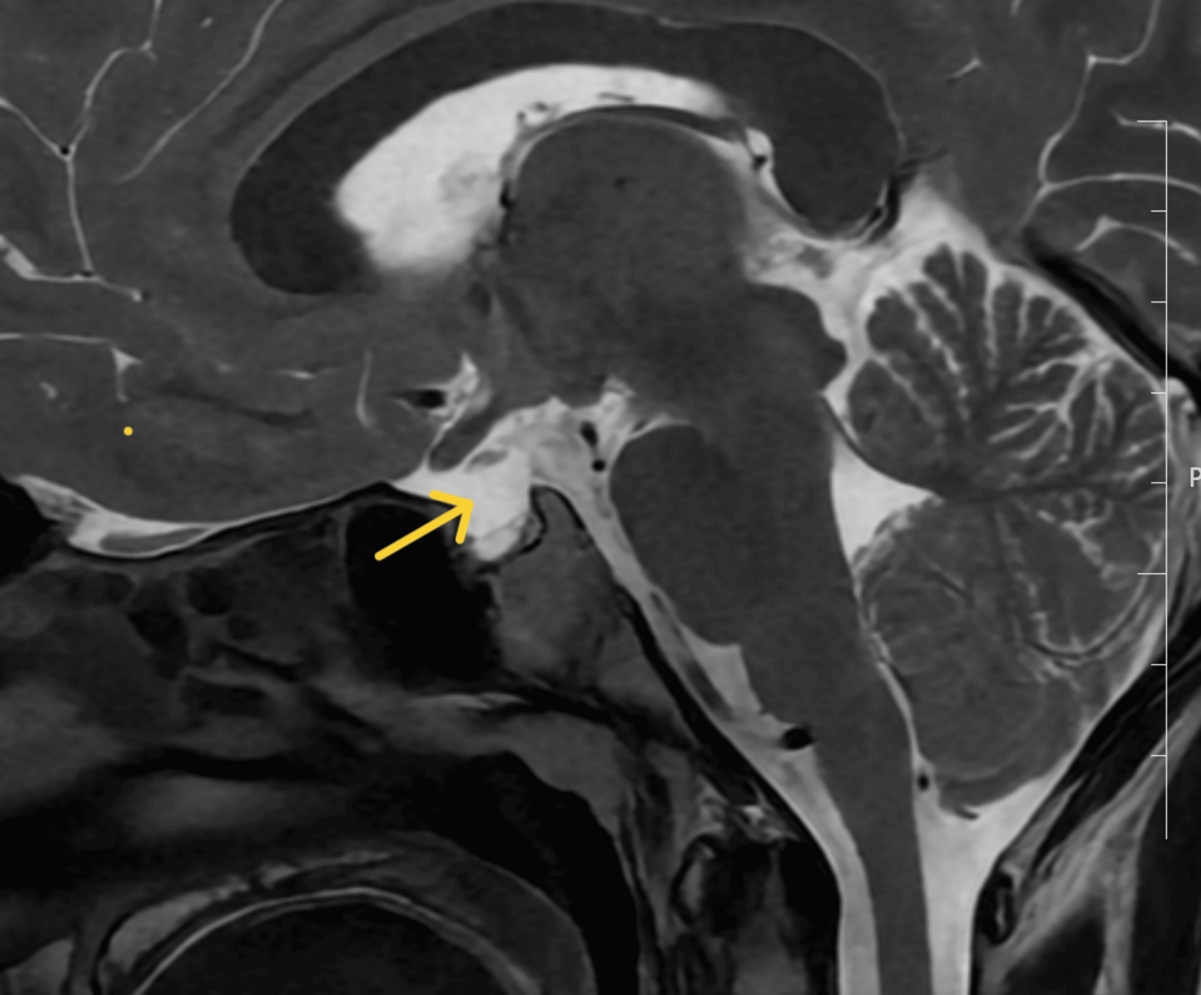
Sagittal T2-weighted MRI of sella turcica showing absent pituitary stalk (yellow arrow) of Case 2

The patient’s complete blood count, liver and kidney function tests, electrolyte levels, calcium profile, and IgA tissue transglutaminase levels (coeliac serology) were normal (Table 1). Hormonal profile (Table 2) showed GHD, central hypothyroidism, hypogonadotropic hypogonadism, and hypocortisolism. His bone age was 13 years and 6 months, indicating a delay in bone age. CEMRI of the sella turcica showed an ectopic posterior pituitary (bright spot) with a hypoplastic anterior pituitary and absent pituitary infundibulum (Figure 3 and Figure 4).

On the basis of this profile, a diagnosis of PSIS with multiple pituitary hormone deficiency (GHD due to low insulin-like growth factor-1 (IGF-1), central hypothyroidism due to low free T4 and normal TSH, and adrenal insufficiency due to low ACTH) was made. The relationship between serum TSH and free T4 is log-linear. Therefore, small changes in serum free T4 levels lead to large changes in TSH. In the above case scenario, even in the presence of low free T4, serum TSH is still in the normal range, which indicates insufficient response, and hence, the patient was diagnosed to have central hypothyroidism. The patient was treated with hydrocortisone (7.5 mg (10 mg/m2) daily) and then levothyroxine (75 mcg), followed by recombinant human growth hormone (0.19 mg/kg/week), and scheduled for a follow-up after 3 months.

Case 3

A 22-year-old male presented with the chief complaint of poor development of secondary sexual characteristics. He was born from a non-consanguineous marriage and was born at term via vaginal breech delivery with no unfavourable prenatal or postnatal outcomes. He had no history of seizures, headaches, vomiting, or other neurological impairment and no history of anosmia, visual deficits, hearing loss, head trauma, testicular trauma, mumps, or chronic systemic illness. There was no family history of any similar illness. General examination showed the presence of bilateral gynecomastia. Physical examination showed that his height and weight were 167 cm (10th-25th percentile) and 75 kg, respectively, with an upper-to-lower segment ratio of 0.81, an arm span of 165 cm, and a body mass index (BMI) of 26.9 kg/m2. The bilateral testicular volume was determined to be 4 ml. The stretched penile length was 5.5 cm, which was less than -2.5 SD, and SMR staging indicated Tanner stage 1. The systemic examination was normal.

**Figure 5:**
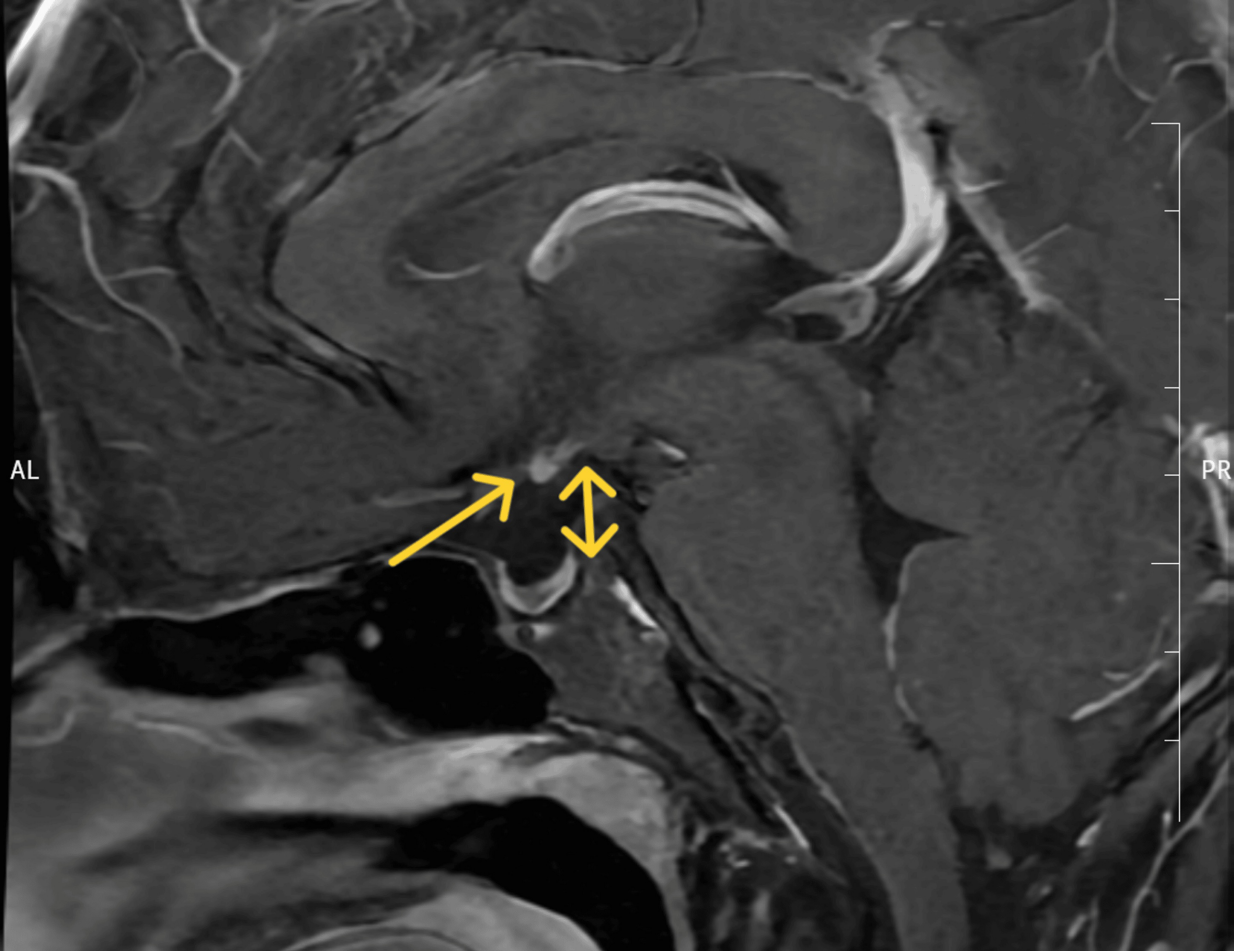
T1-weighted contrast enhanced MRI of sella turcica showing an ectopic posterior pituitary (yellow arrow) and absent pituitary infundibulum in Case 3

The patient’s complete blood count, electrolyte and calcium profile, and renal and liver function tests were normal (Table 1). The hormonal profile (Table 2) revealed hypogonadotropic hypogonadism. CEMRI showed an ectopic posterior pituitary with a hypoplastic anterior pituitary and absent pituitary infundibulum (Figure 5).

Further evaluation showed that he had central hypothyroidism with ACTH deficiency. Therefore, a diagnosis of PSIS with multiple pituitary hormone insufficiencies (GHD, hypogonadotropic hypogonadism, central hypothyroidism, and adrenal insufficiency) was made. The patient was started on hydrocortisone 12.5 mg daily, levothyroxine 75 mcg, and testosterone injection (250 mg) monthly, and scheduled for a follow-up after 3 months.

## Discussion

PSIS is one of the rare causes of hormonal dwarfism. The diagnosis of PSIS is based on radiological findings and supported by clinical and hormonal evaluation. Initially, pituitary stalk damage due to perinatal asphyxia and breech delivery was believed to be the underlying mechanism of PSIS, but current evidence emphasizes the involvement of mutations in the genes associated with hypothalamic-pituitary development (*PIT1*, *PROP1*, *LHX3*/*LHX4*, *PROKR2*, *OTX2*, *TGIF*, *HESX1*, *ROBO1*, and *GPR161*) [16-18]. Mutations in the homeobox gene *HESX1 *are commonly associated with PSIS, which is characterized by septo-optic dysplasia, isolated GHD or multiple hormone deficiencies (including central diabetes insipidus), and delayed puberty [19,20]. An *OTX2 *mutation is associated with anterior pituitary hypoplasia and ectopic posterior pituitary along with severe ocular malformation, including anophthalmia [19,21]. An *LHX3 *mutation can present with hypoplastic anterior pituitary with no abnormal posterior pituitary or midline structure [22,23,24]. The majority of the patients with *LHX3 *mutation present with restricted neck rotation due to a short, rigid cervical spine. The most common presentation of PSIS is short stature; however, it can also present as hypogonadism, delayed puberty, hypocortisolism, hypothyroidism, and hyperprolactinemia.

The short stature in PSIS is caused by GHD, thyroxine deficiency, and cortisol deficiency in isolation or in combination. Growth hormone and IGF1 stimulate the proliferation and maturation of chondrocytes in the epiphyseal growth plate and the differentiation of pre-chondrocytes into chondrocytes, leading to the formation of new cartilage tissue, which is subsequently replaced by bone tissue through endochondral ossification, resulting in linear growth. Thyroid hormone promotes angiogenesis, mineralization, and chondrocyte proliferation and terminal differentiation. Specifically, the development of hypertrophic chondrocytes depends on thyroid hormone levels. Thyroid hormone deficiency causes growth retardation due to the dysregulation of chondrocyte proliferation, mineralization, and angiogenesis [25,26]. Glucocorticoid deficiency causes growth failure by decreasing growth hormone secretion owing to a decreased expression of growth hormone-releasing hormone (GHRH) and growth hormone secretagogue receptors [27].

MRI is a crucial diagnostic tool for PSIS. On MRI, PSIS is characterized by a hypoplastic or absent anterior pituitary, lack of hyperintense posterior pituitary within the sella turcica, and absence or thinned-out pituitary infundibulum [6]. However, MRI presentation of PSIS may vary, regarding the height of the anterior pituitary (which can be absent to normal), the posterior pituitary gland appearance (ectopic at the base of the hypothalamus or along the pituitary stalk, absent, or normal), and the pituitary stalk (interrupted, thin, absent, or normal).

PSIS showed a male preponderance in most published case series [1,28-34], which is consistent with our data. However, there is usually a selection bias in these cases because parents bring male children for developmental evaluation more often than girls. The most prevalent hormone deficiency in patients with PSIS is growth hormone deficiency (100%), followed by gonadotropin deficiency (86.52%), TSH insufficiency (79.78%), and ACTH deficiency (75.28%), as observed in the study of Zhang et al. [35]. The cornerstone of treatment for most PSIS cases is early hormone replacement. This promotes the development of secondary sexual traits and adult height at a normal or nearly normal rate. The three cases in our case series were deficient in growth hormone, TSH, and ACTH. An LH/FSH deficit may manifest early in minipuberty or later in peripuberty. Thus, gonadal evaluation should be performed on a regular basis in patients with PSIS.

Patients with PSIS sometimes present with additional extra-pituitary symptoms. Midline abnormalities may affect the cerebrovascular system and eye structure. Furthermore, extracerebral abnormalities of the skin, heart, and limbs are observed. It is imperative to perform routine examinations of the cardiovascular, ophthalmological, and neurological systems [36]. There are several unknown genetic or antenatal/perinatal causes of PSIS, which presumably contribute to its diverse clinical, biological, and radiological presentation.PSIS may present as an isolated or multiple pituitary hormone deficiency, with variable clinical severity. PSIS was formerly identified by short stature (growth hormone deficiency) and varying degrees of additional symptoms of anterior and/or posterior pituitary dysfunction. It has been reported more recently that not all patients with PSIS exhibit aberrant secondary sexual features and short stature. Patients may, in fact, seem to develop normally as adults with only slight variations in the pituitary hormone profile [37].

## Conclusions

This case series highlights the heterogeneous presentation of PSIS, with short stature being the most consistent early clinical sign. The variability in age at diagnosis underscores the diagnostic challenge, necessitating a high index of suspicion, especially in cases of unexplained growth failure. Growth hormone deficiency was the most prevalent hormonal abnormality observed in this case series, often accompanied by thyroid hormone deficiency. Patients with PSIS presenting before epiphyseal fusion can attain normal height with proper treatment and follow-up. Notably, timely hormone replacement therapy led to significant improvements in growth and pubertal development, reinforcing the importance of early endocrine intervention. Given the rarity of PSIS, with the variable age of presentation and the wide spectrum of manifestations depending on the associated hormone deficiency, early diagnosis, close monitoring, and timely treatment may improve the prognosis in these patients.
